# The First Identified Nucleocytoplasmic Shuttling Herpesviral Capsid Protein: Herpes Simplex Virus Type 1 VP19C

**DOI:** 10.1371/journal.pone.0041825

**Published:** 2012-08-22

**Authors:** Lei Zhao, Chunfu Zheng

**Affiliations:** 1 State Key Laboratory of Virology, Wuhan Institute of Virology, Chinese Academy of Sciences, Wuhan, China; 2 Division of Scientific Research, Northwest A&F University, Yangling, Shaanxi, China; University of Minnesota, United States of America

## Abstract

VP19C is a structural protein of herpes simplex virus type 1 viral particle, which is essential for assembly of the capsid. In this study, a nuclear export signal (NES) of VP19C is for the first time identified and mapped to amino acid residues 342 to 351. Furthermore, VP19C is demonstrated to shuttle between the nucleus and the cytoplasm through the NES in a chromosomal region maintenance 1 (CRM1)-dependent manner involving RanGTP hydrolysis. This makes VP19C the first herpesviral capsid protein with nucleocytoplasmic shuttling property and adds it to the list of HSV-1 nucleocytoplasmic shuttling proteins.

## Introduction

Herpes simplex virus type 1 (HSV-1), a typical alphaherpesvirus, causes a spectrum of diseases, including herpes labialis, herpes keratitis, and herpes encephalitis, which can be lethal [1]. VP19C is the HSV-1 structural protein known to localize in the nucleus of infected cells and essential for capsid formation [2], [3]. Previous studies have demonstrated that N-terminus of VP19C is required for nuclear import of VP19C [4].

Similar to nuclear import, export of a protein from nucleus depends on the presence of a specific nuclear export signal (NES) [5]. An increasing number of viral proteins, including human immunodeficiency virus type 1 (HIV-1) Rev [6], human T-cell lymphotropic virus type 1 (HTLV-1) Rex [7], influenza virus NS1 [8], HSV-1 ICP27 [9], γ134.5 [10], Epstein-Barr virus (EBV) SM [11], bovine herpesvirus-1 (BHV-1) VP8 [12], and BHV-1 BICP27 [13] have been found to contain a leucine-rich NES sequence enabling the protein to shuttle between the nucleus and the cytoplasm.

## Materials and Methods

### Plasmids construction

All enzymes used for cloning procedures were purchased from Takara (Dalian, China) except for T4 DNA ligase from New England Biolabs (MA, USA). The open reading frame of VP19C gene of HSV-1 F strain was amplified from the HSV-BAC DNA (pYEbac102) and cloned into *Eco*RI/*Bam*HI-digested pEYFP-N1 (Clontech, CA, USA) to yield plasmid VP19C-EYFP as described previously [14]. Similarly, other plasmids expressing of mutant VP19C were constructed and confirmed by sequencing. All primers used in this study are available on request. pRev-NES-EGFP was a generous gift from Dr. Gillian Elliott [15] and was used as the positive control for leptomycin B (LMB) treatment. Ran-Q69LECFP was constructed from pGEX6p-1 Q69L Ran as described previously [13].

### Transfection, fluorescence microscopy and LMB treatment

Transfection and living cells fluorescence microscopy experiments were performed as described previously [13], [16]. For LMB treatment, 24 h after transfection, LMB (Sigma, MO, USA) was added to the culture medium at a final concentration of 10 ng/ml for 4 h as described previously [13], [16]. The live cells were then examined using fluorescence microscopy (Zeiss, Jena, Germany). All the photomicrographs were taken under a magnification of 400×. Each photomicrograph represents the vast majority of the cells with similar subcellular localization. Both fluorescent images of EYFP and ECFP fusion proteins were presented in pseudocolor, green and red, respectively. Images were processed with Adobe Photoshop.

### Heterokaryon assays

To analyze the property of VP19C to shuttle between the nucleus and cytoplasm, interspecies heterokaryons of COS-7 and mouse NIH 3T3 cells were performed as described previously [12], [16]. For the LMB treatment, cells were incubated at 10 ng/ml for 4 hrs before and after the cell fusion. COS-7 cells were firstly infected with HSV-1 at MOI of 2. At 12 hrs after infection, the HSV-1 infected COS-7 cells were mixed with 3T3 cells and subjected to the heterokaryon assay.

## Results and Discussion

**Figure 1:**
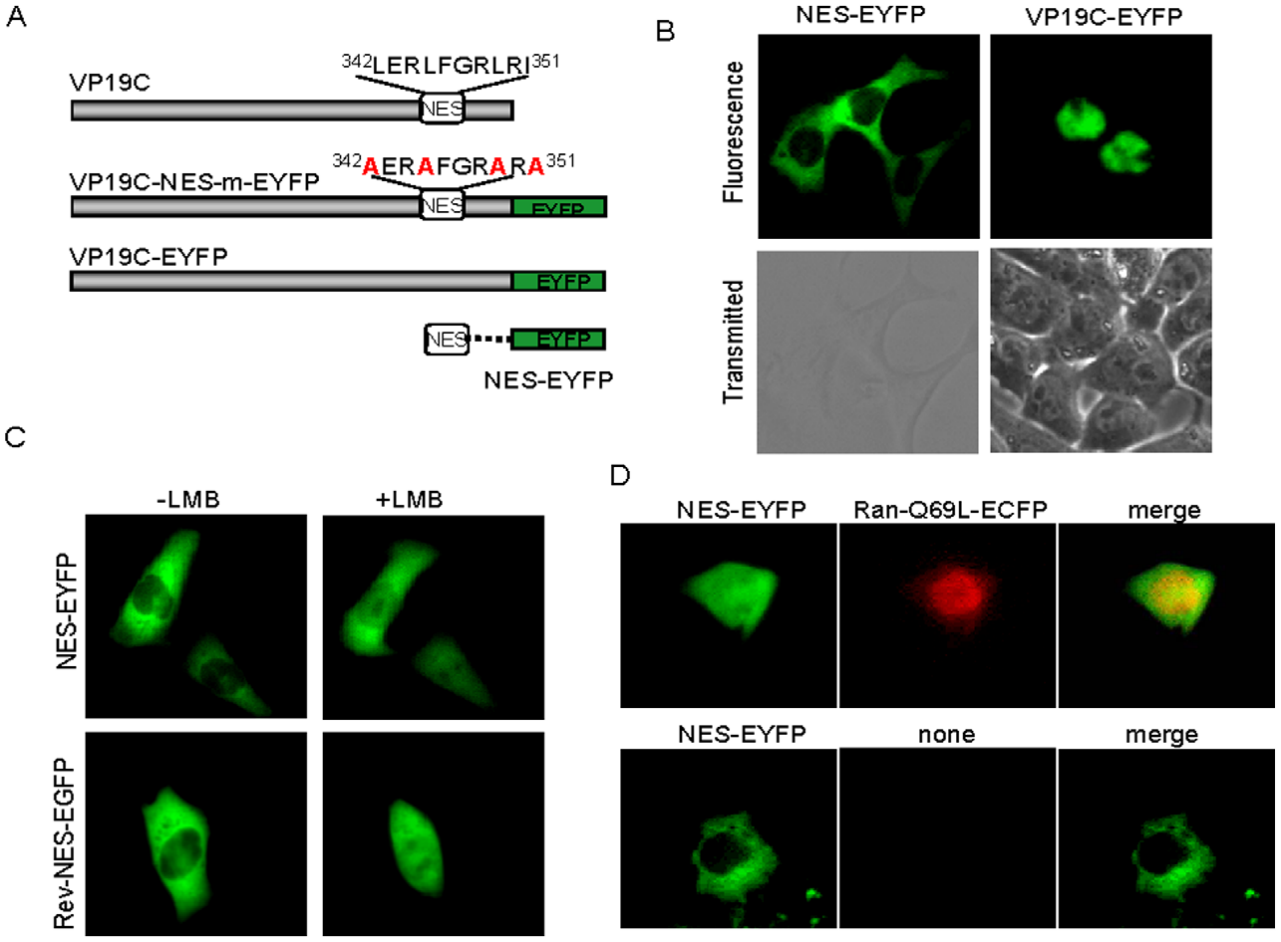
Characterization of the NES and the nuclear export mechanism of the VP19C protein. (A) Schematic diagram of wild-type VP19C and the putative NES fused with EYFP. (B) Subcellular localization of NES-EYFP and VP19C-EYFP. (C) The NES of VP19C mediated the nuclear export of EYFP via CRM1 dependent pathway. COS-7 cells were transiently transfected with plasmids encoding NES-EYFP and Rev-NES-EYFP (positive control). The cells were incubated in the absence or presence of 10 ng/ml LMB 24 h after transfection. (D) The NES of VP19C mediated the nuclear export of EYFP via RanGTP. COS-7 cells were co-transfected with pNES-EYFP with or without pRan-Q69L-ECFP. Both fluorescent images of EYFP and ECFP fusion proteins were presented in pseudocolor, green and red, respectively. Each image is representative of the vast majority of the cells observed.

An examination of the C-terminal region of VP19C revealed that it contained a leucine-rich motif, ^342^LERLFGRLRI^351^ (Fig. 1A), resembling the most prevalent NES consensus (ΦX_2–3_ΦX_2–3_ΦXΦ [Φ represents any hydrophobic amino acids, such as leucine, isoleucine, valine, tryptophan, or methionine, whereas×represents any amino acids]) [17], which was speculated to be a potential NES. A DNA fragment containing the putative NES sequence, ^342^LERLFGRLRI^351^, was synthesized and then ligated to the 5′ terminus of enhanced yellow fluorescent protein (EYFP) DNA sequence, yielding NES-EYFP (Fig. 1A) as described previously [13], [16]. A plasmid encoding VP19C-EYFP was constructed previously [14]. As expected, VP19C-EYFP targeted to the nucleus (Fig. 1B). However, the NES of VP19C directed EYFP exclusively to the cytoplasm (Fig. 1B), indicating that the NES was sufficient to mediate the nuclear export of EYFP, and the sequence, ^342^LERLFGRLRI^351^, possessed authentic and transferable NES activity and was a functional NES. This is the first report of a functional NES within capsid protein VP19C.

Leucine-rich NES has been identified in an increasing number of cellular and viral proteins executing quite heterologous biological functions. Most studies pertaining to nuclear export have implicated the chromosome region maintenance 1 (CRM1) in facilitating the export of NES-containing proteins. An antibiotic compound, LMB, has been found to specifically inhibit CRM1-mediated nuclear export [18], [19]. It alkylates a cysteine residue in the nuclear export receptor CRM1 and thereby blocks binding and translocation of target proteins [19]. To investigate the nuclear export mechanism of VP19C NES, LMB treatment was performed after transfection of pNES-EYFP and positive control pRev-NES-EGFP [15] as described previously [16]. As shown in Fig. 1C, NES-EYFP was distributed throughout the cell after LMB treatment. LMB increased NES-EYFP nuclear accumulation, suggesting that the export of VP19C NES was CRM1 dependent. Similarly, the LMB treatment could completely abolish the nuclear export of positive control Rev-NES-EGFP containing classic leucine-rich NES [19], [20]. Taken together, these results suggested that this NES might physically interact with CRM1 and the functional NES mediated the nuclear export of VP19C through a CRM1-dependent pathway.

CRM1 cannot leave the nucleus with its cargo by itself but needs to be associated with Ran. In the nucleus, direct binding of RanGTP to CRM1 stabilizes the association of the receptor with NES cargo, the resulting trimeric export complex can then undergo translocation through the nuclear pore complex (NPC) [21], [22]. To further explore the nuclear export mechanism of VP19C, a mutated Ran protein, Ran-Q69L, which was deficient in GTP hydrolysis, was introduced to determine whether Ran was required for the nuclear export of VP19C [23], [24]. Co-transfection of NES-EYFP with Ran Q69L-ECFP revealed that over expression of Ran Q69L significantly abolished the nuclear export of VP19C, whereas NES-EYFP alone targeted to the cytoplasm (Fig. 1D), suggesting that the nuclear export activity of the NES was Ran dependent. It's believed that RanGTP and NES-containing proteins bind cooperatively to CRM1 upon formation of a ternary CRM1-RanGTP-NES complex [21].

**Figure 2:**
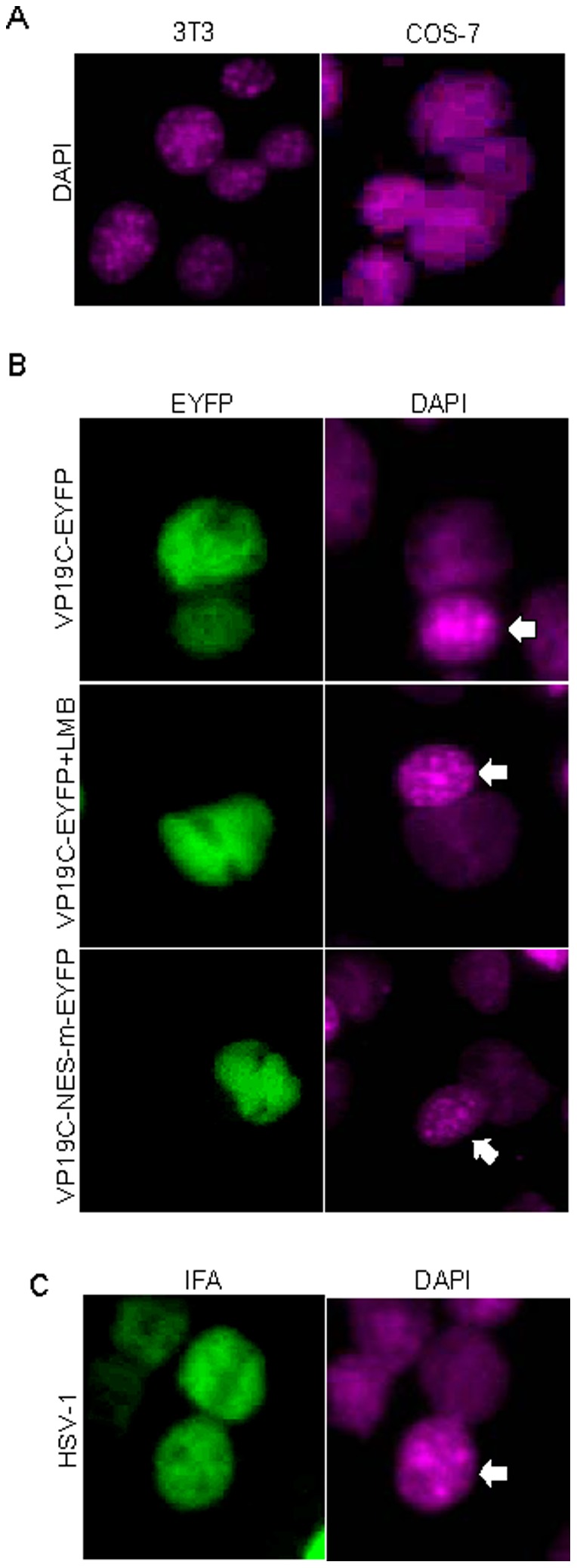
Nucleocytoplasmic shuttling of VP19C. (**A**) **DAPI staining differentiates monkey (COS-7) and murine (3T3) nuclei.** (B) Nucleocytoplasmic shuttling of VP19C via a leucine-rich NES: COS-7 cells were transfected with plasmid VP19C-EYFP or VP19C NES-m-EYFP. 24 hours later, transfected cells were subjected to the interspecies heterokaryon assay. Mouse 3T3 cells were identified by their speckled nuclei when stained with DAPI. (C) Nucleocytoplasmic shuttling of VP19C during HSV-1 infection. COS-7 cells were firstly infected with HSV-1 at MOI of 2. At 12 hour after infection, the HSV-1 infected COS-7 cells were mixed with 3T3 cells and subjected to the heterokaryon assay. Mouse 3T3 cells were identified by their speckled nuclei when stained with DAPI. Each image is representative of the vast majority of the cells observed.

The vast majority of nucleocytoplasmic shuttling proteins described so far possess an NES similar to the leucine-rich NES of the HIV-1 Rev protein [25]. As VP19C contains the similar leucine-rich motif, ^342^LERLFGRLRI^351^, we speculated that VP19C could also shuttle between the nucleus and the cytoplasm. Nucleocytoplasmic shuttling was detected via an interspecies heterokaryon assay as described previously and this assay has been successfully applied to identify a few nucleocytoplasmic shuttling viral proteins in our lab [12], [13], [16], [20]. DAPI staining allowed differentiation between monkey (COS-7) and mouse (3T3) nuclei because monkey cells stained diffusely throughout the nuclei, whereas mouse nuclei stained with a distinctive speckle pattern (Fig. 2A). COS-7 cells were transfected with plasmid expressing VP19C-EYFP overnight to allow synthesis of the protein and subsequently were subjected to the heterokaryons assay with 3T3 cells in the presence of the protein synthesis inhibitor cycloheximide. The subcellular localization of VP19C in the heterokaryons assay was assessed by fluorescence microscopy (Fig. 2B). As expected, VP19C-EYFP was detected in the nuclei of 3T3 (Fig. 2B and Table 1). Since protein synthesis was inhibited, the appearance of VP19C in the nucleus of mouse 3T3 cells suggested that VP19C had shuttled out of the nuclei of the transfected monkey COS-7 cells and been re-imported into the nuclei of untransfected mouse 3T3 cells, suggesting that VP19C was able to shuttle across the nuclear membrane in both directions. However, VP19C-EYFP was not observed in non-fused mouse cells (data not shown).

**Table 1:** Heterokaryon assay cell counts.

Vectors used for transfection or HSV-1 infection^a^	No. of mouse nuclei	
	+^b^	−c
**VP19C-EYFP**	47	3
**VP19C-EYFP+LMB**	2	48
**VP19C-NES-m-EYFP**	2	48
**HSV-1**	47	3

a COS-7 cells were transiently transfected with the various expression vectors, and 24 h later a heterokaryon assay was carried out with NIH 3T3 cells or COS-7 cells were infected with HSV-1, and 12 h later a heterokaryon assay was carried out with 3T3 cells.

b Mouse nuclei were considered positive if they were present in fused heterokaryons of COS-7 cells expressing VP19C-EYFP or mutant proteins and monkey 3T3 cells contained detectable levels of the expressed protein.

c Mouse nuclei were considered negative if they were present in fused heterokaryons of COS-7 cells expressing VP19C-EYFP or mutant proteins and monkey 3T3 cells did not contain detectable levels of the expressed protein.

To determine whether the nucleocytoplasmic shuttling of VP19C is dependent on its association with the export receptor CRM1, the heterokaryons assay for cells expressing VP19C-EYFP fusion protein were performed in the presence of LMB. For the LMB treatment, cells were incubated at 10 ng/ml for 4 hrs before and after the cell fusion. As results, the nucleocytoplasmic shuttling of VP19C-EYFP was significantly inhibited by LMB (Fig. 2B and Table 1). This further demonstrated that the export receptor CRM1 was required for the export of VP19C.

Based on the defined NES in VP19C, VP19C mutant was constructed to substitute leucine and isoleucine residues in the NES with alanine residues (VP19C-NES-m) (Fig. 1A). Analogous mutations in other NES-containing proteins have been reported to prevent the nuclear export [26], [27]. A heterokaryon assay was also performed to determine whether the functional NES is required for the nucleocytoplasmic shuttling of VP19C. As results, VP19C-NES-m did not shuttle from the nuclei of monkey COS-7 cells to the nuclei of murine 3T3 cells (Fig. 2B and Table 1). This indicated that mutations of the leucine and isoleucine residues within the NES abrogated the ability of the VP19C to shuttle between the nucleus and the cytoplasm. To further investigate whether VP19C shuttles during HSV-1 infection, COS-7 cells were infected with HSV-1 at a multiplicity of infection (MOI) of 2. Twelve hours later, HSV-1 infected cells were mixed with 3T3 cells and subjected to the heterokaryon assay in the presence of the protein synthesis inhibitor cycloheximide. The subcellular localization of VP19C cells were analyzed 12 hrs after co-culture of the mixed cells by an immunofluorescence assay with mouse monoclonal antibody mAb02040 raised against purified VP19C [4]. As results, VP19C shuttled to the nuclei of mouse 3T3 cells (Fig. 2C and Table 1). Taken together, these data suggested that VP19C was a nucleocytoplasmic shuttling protein exported from the nucleus through the NES via a CRM1-independent pathway.

VP19C protein has been previously shown to bind viral DNA in a nonspecific fashion and may play a role in anchoring the DNA to the capsid [28], [29]. It is reported that correct transport of the component proteins to the site of capsid assembly is an important function of VP19C [4]. To ensure accurate cellular functioning, the spatial distribution of proteins needs to be delicately regulated and coordinated. The nucleocytoplasmic shuttling capability of VP19C suggests that besides essential for the assembly of the capsid shell structure in the nucleus, VP19C may also function as a carrier to transport cytoplasmic capsid protein, such as VP 23 [2], [30], [31], to the nucleus and facilitate the assembly of the capsid.

In conclusion, a leucine-rich NES ^342^LERLFGRLRI^351^ was identified in VP19C, and it could mediate the nuclear export of a heterologous, non-shuttling protein EYFP in an LMB-sensitive and Ran-dependent manner. Furthermore, VP19C was demonstrated to shuttle between the nucleus and the cytoplasm in VP19C transfected and HSV-1 infected cells via a CRM1 dependent pathway. This made VP19C the first herpesviral capsid protein with nucleocytoplasmic shuttling property and added it to the list of HSV-1 nucleocytoplasmic shuttling proteins.
